# Lysosomal calcium loading promotes spontaneous calcium release by potentiating ryanodine receptors

**DOI:** 10.1016/j.bpj.2023.06.007

**Published:** 2023-06-15

**Authors:** Zhaozheng Meng, Rebecca A. Capel, Samuel J. Bose, Erik Bosch, Sophia de Jong, Robert Planque, Antony Galione, Rebecca A.B. Burton, Alfonso Bueno-Orovio

**Affiliations:** 1 Department of Pharmacology, University of Oxford, Oxford, United Kingdom; 2 Department of Mathematics, Vrije Universiteit Amsterdam, Amsterdam, the Netherlands; 3 Department of Computer Science, University of Oxford, Oxford, United Kingdom

**Keywords:** lysosomes, calcium handling, β-adrenergic stimulation, modeling and simulation

## Abstract

Spontaneous calcium release by ryanodine receptors (RyRs) due to intracellular calcium overload results in delayed afterdepolarizations, closely associated with life-threatening arrhythmias. In this regard, inhibiting lysosomal calcium release by two-pore channel 2 (TPC2) knockout has been shown to reduce the incidence of ventricular arrhythmias under β-adrenergic stimulation. However, mechanistic investigations into the role of lysosomal function on RyR spontaneous release remain missing. We investigate the calcium handling mechanisms by which lysosome function modulates RyR spontaneous release, and determine how lysosomes are able to mediate arrhythmias by its influence on calcium loading. Mechanistic studies were conducted using a population of biophysically detailed mouse ventricular models including for the first time modeling of lysosomal function, and calibrated by experimental calcium transients modulated by TPC2. We demonstrate that lysosomal calcium uptake and release can synergistically provide a pathway for fast calcium transport, by which lysosomal calcium release primarily modulates sarcoplasmic reticulum calcium reuptake and RyR release. Enhancement of this lysosomal transport pathway promoted RyR spontaneous release by elevating RyR open probability. In contrast, blocking either lysosomal calcium uptake or release revealed an antiarrhythmic impact. Under conditions of calcium overload, our results indicate that these responses are strongly modulated by intercellular variability in L-type calcium current, RyR release, and sarcoplasmic reticulum calcium-ATPase reuptake. Altogether, our investigations identify that lysosomal calcium handling directly influences RyR spontaneous release by regulating RyR open probability, suggesting antiarrhythmic strategies and identifying key modulators of lysosomal proarrhythmic action.

## Significance

Delayed afterdepolarizations arising from spontaneous RyR calcium release are an important risk factor for arrhythmogenesis. Inhibiting lysosomal calcium release by TPC2-KO reduces the propensity for ventricular arrhythmias. However, understanding downstream effects of lysosomal calcium release on spontaneous RyR release is lacking. Understanding lysosomes as arrhythmia sources requires alternative approaches to controlled laboratory techniques: these restrain variability experimentally and statistically. Our study presents two methodological novelties by focusing on calibration with experimental findings using a population of biophysically detailed models and incorporating lysosomal mechanisms. Lysosomal calcium handling promotes RyR spontaneous release by elevating RyR open probability. Blocking lysosomal function uncovers an antiarrhythmic strategy. Lysosome-release proarrhythmic risk is determined by synergistic enhancements of lysosomal uptake with RyR release or L-type calcium current.

## Introduction

Disruption in calcium signaling underlies many cardiac disorders, including contractile dysfunction and rhythm disorders (1,2,3,4). In cardiomyocytes, the process of excitation-contraction coupling links depolarization of the plasma membrane to muscle fiber contraction (5). Membrane depolarization in response to the arrival of an action potential leads to the activation of voltage-gated L-type calcium channels located within the sarcolemma and transverse (t-)tubules. The resulting inward calcium current (I_CaL_) causes an increase in calcium concentration within the subsarcolemmal junctional space, located between the surface membrane and the sarcoplasmic reticulum (SR). This elevation in junctional calcium activates ryanodine receptors (RyRs), which then release calcium from the SR into the cytosol. If sufficient RyRs are activated, this process of calcium-induced calcium release (CICR) leads to a cellular calcium transient characterized by an initial increase in cytosolic calcium followed by a decrease resulting from the combination of RyR closure, reuptake of calcium into SR via the SR calcium-ATPase (SERCA), and extrusion from the cell via the sodium-calcium exchanger (NCX). Calcium transients are linked to cell contraction due to the binding and activation of cytosolic calcium to troponin on the muscle filaments (5). Tight regulation of cellular calcium is therefore essential in cardiac cells to maintain healthy rhythm and contractility.

Calcium in cardiomyocytes is primarily buffered within the SR. However, other organelles including lysosomes and mitochondria may also act as calcium stores and influence calcium handling (6,7,8). In addition to β-adrenergic signaling, calcium signaling undergoes regulation via multiple alternative pathways and messengers, including nicotinic acid adenine dinucleotide phosphate (NAADP), inositol-1,4,5-trisphosphate, and cyclic ADP ribose (3,6,7,8,9).

The involvement of lysosomes in cardiac disorders has long been recognized. In the 1960s, higher numbers of lysosomes were observed in the atrial tissue of chronically diseased hearts from human patients (10), and lysosomes were increased in canine atria after introduction of atrial septal defects (11). Multiple studies have since demonstrated that lysosomal dysfunction can be linked to cardiac disorders including arrhythmia and heart failure (9,12,13,14). Lysosomal calcium release occurs primarily through the opening of type 2 two-pore channels (TPC2) after activation by NAADP (13,15,16,17,18). Transmission electron microscopy has shown that, in cardiomyocytes, lysosomes are located within close proximity to the SR, mitochondria, and t-tubules, and may form signaling microdomains involving membrane contact sites with these organelles (19). Release of lysosomal calcium via TPC2 in response to NAADP enhances RyR calcium release from the SR (20), and inhibition of the NAADP pathway has been shown to be protective against β-adrenergic-induced arrhythmias (21). Furthermore, lysosomes have been shown to generate oscillating calcium currents that can trigger potentially lethal cardiac arrhythmias, and these oscillations can also be suppressed by the inhibition of NAADP (22). Despite these observations, the role of lysosomal calcium signaling in cardiomyocytes has been relatively understudied and lysosomes are not included in current mathematical models of cardiac calcium handling (23,24). This severely constrains our understanding of their possible proarrhythmic role and our capability to suggest appropriate antiarrhythmic therapies.

While separate components of calcium signaling within cardiomyocytes can be investigated experimentally in isolation, mathematical modeling enables comprehensive predictive studies to be performed consistently by integrating these components (23,24). Models of cardiac excitability have several benefits compared with physiological experiments. Biological experiments are subject to the combined limitations of loss of variability, for example, through the averaging of data, as well as limited sample size and throughput (25,26,27). Mathematical models based purely on experimental data can therefore of course also suffer from the loss of individuality resulting from the averaging of biological data (25). However, the use of more recent advances in approaches for studying variability, such as experimentally calibrated population of models frameworks, enables such biological variability to be incorporated into models of cardiac electrophysiological signaling (26,27). Here, we modify an existing model of mouse ventricular calcium handling by Morotti et al. (24) to include a lysosomal compartment based on experimental data on spatial organization and lysosomal action. In our updated model we adopt a population of models-based approach to account for variability in electrophysiology and calcium handling (26,27). This model was used to investigate mechanisms of lysosomal calcium handling in arrhythmogenesis and RyR spontaneous release under conditions of calcium overload (hypercalcemia and fast pacing) in conjunction with β-adrenergic stimulation. Our modeling data support the hypothesis that lysosomes can act as an additional intracellular calcium store, providing an additional transport for intracellular calcium, and can influence the driving force for spontaneous calcium release from the SR to the junctional space. Identifying new targets in arrhythmic processes is therefore of great importance and has potentially high clinical value.

## Materials and methods

### Mathematical modeling and simulation studies

The model by Morotti et al. (24) was used as the baseline mathematical model for our investigations because of matching species and the inclusion of β-adrenergic response and CaMKII signaling. A lysosomal calcium compartment was formulated into the model to account for lysosomal calcium handling, as detailed next. The simulated results matched calcium transients and action potentials in the experimental results (13). Each simulation was run for 150 beats at 1 Hz pacing rate to allow the system to enter steady state, and all reported biomarkers were calculated on the last beat, unless otherwise specified. NAADP concentration (*[NAADP]*) was used as input parameter to regulate TPC2 release, with values of 1 nM for control (CTRL) and 15 nM to simulate nearly fully open probability under either exposure to isoprenaline (ISO) or NAADP-AM (cell permeant analog of NAADP). An ISO ligand value of 100 nM was used in the simulated ISO protocols, which matched the predefined ISO responses of the Morotti et al. model with the magnitude of β-adrenergic response observed in our experimental data.

Additional details relating to the model specifics and experimental information from the Morotti et al. model are provided below (24). Further detailed information can be obtained from the supplorting material in Morotti et al. (24). The Morotti et al. ventricular cardiomyocyte model is based on the framework of the Soltis and Saucerman rabbit model by species-specific changes in cell size and geometry, expression and kinetics of ion channels, and calcium and sodium handling (24,28). Simulated calcium transients recapitulate experimental results of systolic and diastolic calcium and time to 50% calcium transient decay in different pacing frequencies. Simulated action potentials recapitulate waveform morphology and dynamics, with small adaptation of action potential duration to pacing frequency. In summary, simulated traces, predicting mild prolongation under β-adrenergic stimulation in action potential, decrease in diastolic calcium, increase in calcium transient amplitude, and faster calcium transient decay, agree with validation experimental data.

In the Morotti et al. model, RyR open probability is increased by ISO application, and results in a twofold increase under maximal protein kinase A (PKA) phosphorylation. When phospholamban phosphorylation is at its maximum, it results in a 55% decrease in SERCA forward mode. β-Adrenergic stimulation is performed by ISO at 100 nM, leading to a 56% increase in L-type current availability. Under full PKA activation, the combined effect produces a 55% increase in the peak and a twofold increase in calcium influx of L-type current.

### Modified ventricular cell model with lysosome compartment

**Figure 1:**
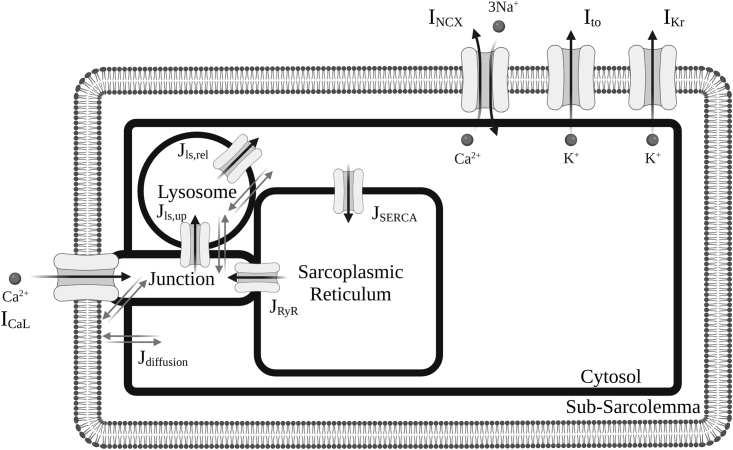
Schematic diagram of modified ventricular cell model with lysosome compartment. Cellular electrophysiology of mouse ventricular cardiomyocyte is represented in the existing Morotti et al. (24) model with β-adrenergic stimulation and CaMKII signaling. Only the main ion channels varied in a population of models approach in this study are shown. A more complete overview of the model can be found in Morotti et al. (24).

Fig. 1 illustrates the integration of the new lysosomal compartment into the Morotti et al. model. Based on organelle localization and lysosome action in experimental findings (13,18,19,29), the lysosomal compartment was included in the model as an additional calcium pool, incorporating calcium diffusion with the neighboring junctional and cytosolic subspaces, as well as a calcium loading channel (CLC) between the junctional and lysosomal subspaces, and lysosomal calcium release through TPC2 into the cytosolic subspace.

**Figure 2:**
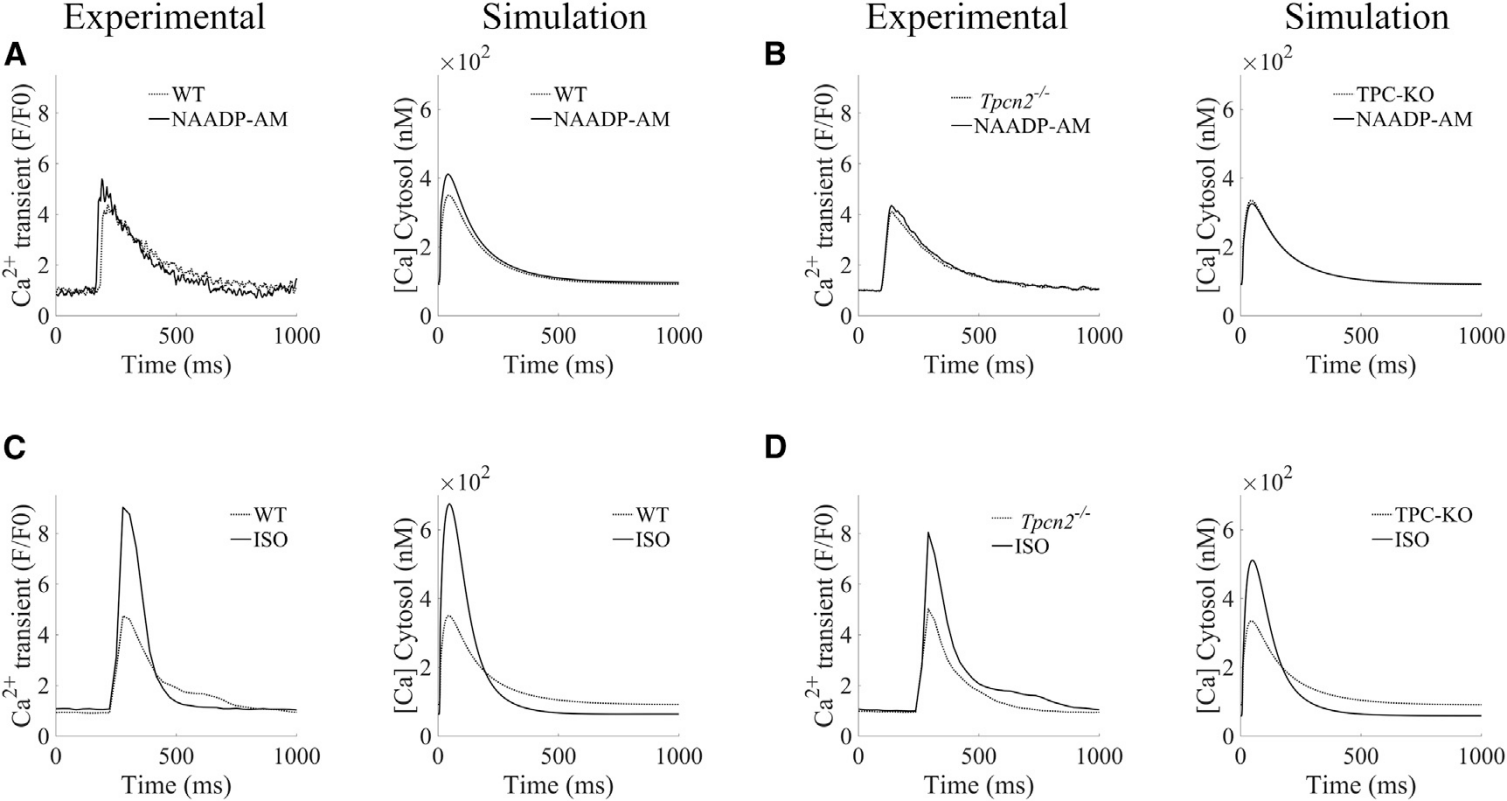
Validation of lysosomal calcium handling model against pharmacological protocols. (*A* and *B*) Calibration of Ca^2+^ transients under increased lysosomal release by NAADP-AM (WT versus TPC2-KO, respectively). (*C* and *D*) Validation of Ca^2+^ transients under β-adrenergic stimulation by ISO (WT versus TPC2-KO, respectively).

The lysosomal calcium inward flux was modeled as a calcium loading channel between the junctional and lysosome subspaces. The lysosomal calcium release through TPC2 was modeled as a lysosomal calcium efflux from the lysosome compartment into the cytosolic subspace. These are, respectively, given by:

$$J_{ls,up}=j_{clc}\cdot P_{O}\cdot \left(C_{ls}-C_{j}\right)+j_{clc,leak}\cdot \left(C_{ls}-C_{j}\right)\text{,}$$

$$J_{ls,rel}=j_{tpc}\cdot P_{O}\cdot \left(C_{ls}-C_{i}\right)+j_{tpc,leak}\cdot \left(C_{ls}-C_{i}\right)\text{,}$$

where *j*_*clc*_ and *j*_*tpc*_ denote the maximal CLC uptake and TPC2 release flux densities into the lysosomal and cytosolic subspaces; *C*_*ls*_, *C*_*j*_, and *C*_*i*_ are the calcium concentrations in the lysosomal, junctional, and cytosolic subspaces, respectively; *j*_*clc, leak*_ and *j*_*tpc, leak*_ denote the leak rates of calcium through the CLC and TPC2 channels to the junctional and cytosolic subspaces, respectively; and *P*_*O*_ represents the open probability of the lysosomal uptake and release channels. Based on previous experimental data (13,18), we adopt the formulation of Penny et al. (30) to describe the open probably of TPC2 channels. This is given by:

$$P_{O}=P_{Omax}\cdot \exp\left(\frac{{-\left(\log\left(\left[NAADP\right]\right)-\log\left(P_{Omean}\right)\right)}^{2}}{2\cdot P_{Osd}^{2}}\right)\text{,}$$

so that $P_{O}$ follows a Gaussian distribution on *[NAADP]*, *P*_*Omax*_ denotes its maximum open probability, and *P*_*Omean*_ and *P*_*Osd*_ represent the mean and standard deviation of the distribution. For CLC channels, given the lack of experimental datasets, we adopted an analogous formulation to TPC2 channels. Such an assumption correctly recapitulated our calcium transient data under CTRL, NAADP-AM, and ISO protocols (see Fig. 2). This modeling assumption can nevertheless be easily revisited, shall data sets on lysosomal calcium loading become available.

The inclusion of the new lysosomal calcium fluxes then yields the following system of ordinary differential equations for conservation of mass of calcium ions:

$${\dot{C}}_{ls}=J_{ls,j}\frac{C_{j}-C_{ls}}{V_{ls}}-J_{ls,up}+J_{ls,i}\frac{C_{i}-C_{ls}}{V_{ls}}-J_{ls,rel}\text{,}$$

$${\dot{C}}_{j}=J_{j,sl}\frac{C_{sl}-C_{j}}{V_{j}}-I_{Ca,j}-J_{CaB,j}+J_{RyR}\frac{V_{SR}}{V_{j}}+J_{RyR,lk}\frac{V_{c}}{V_{j}}+J_{ls,up}\frac{V_{ls}}{V_{j}}+J_{ls,j}\frac{C_{ls}-C_{j}}{V_{j}}\text{,}$$

$${\dot{C}}_{i}=J_{sl,i}\frac{C_{sl}-C_{i}}{V_{i}}-J_{SERCA}-J_{CaB,i}+J_{ls,rel}\frac{V_{ls}}{V_{i}}+J_{ls,i}\frac{C_{ls}-C_{i}}{V_{i}}\text{,}$$

where ${\dot{C}}_{ls}$, ${\dot{C}}_{j}$, and ${\dot{C}}_{i}$ denote the time derivatives of calcium concentrations in the lysosomal, junctional, and cytosolic subspaces, respectively, while *V*_*ls*_, *V*_*j*_, *V*_*SR*_, and *V*_*i*_ represent the volumes of the lysosomal, junctional, SR, and cytosolic subspaces. In the equations above, *I*_*Ca,j*_ denotes the junctional calcium fluxes via calcium currents, *J*_*CaB,j*_ are the junctional calcium buffers, *J*_*RyR*_ and *J*_*RyR,lk*_ the release and leak fluxes through RyRs, respectively, *J*_*SERCA*_ denotes the calcium reuptake fluxes by SERCA, and *J*_*CaB,i*_ the cytosolic calcium buffers. In these equations, $J_{ls,j}$ and $J_{ls,i}$ are the rates of diffusion fluxes between the lysosome calcium pool and junctional or cytosolic subspaces, respectively.

The remaining equations in the Morotti et al. model (24) remained unaltered, including those describing calcium concentrations in the SR and sarcolemmal compartments. Parametrization of the lysosomal compartment is provided in Table S1.

### Experimental data

The experimental data sets include calcium transients and action potentials recordings at 1 Hz under CTRL, NAADP-AM, and ISO protocols in both wild-type (WT) and TPC2-KO mouse ventricular cardiomyocytes, and have been reproduced with permission from the original authors (13,31).

### Cellular variability in electrophysiology and calcium handling

We adopted a population of models approach (26,27) to account for cellular variability in electrophysiology and calcium handling. Such an approach has been especially effective in investigating mechanisms of proarrhythmia, including those associated with intracellular calcium handling (26,27,32,33). Variability was considered by modulating the maximal densities of the main transmembrane ionic currents and intracellular fluxes regulating the AP and calcium handling in the mouse ventricular cardiomyocyte discussed as follows. An initial population of 1000 models, representing 1000 cells with different ionic properties, was generated by means of uniform Latin hypercube sampling of scaling factors (scaling from 0.5- to 2-fold) for the following model components: I_CaL_, as main driver of calcium influx into the cardiomyocyte; SR calcium release flux from RyR (J_RyR_), maximal lysosomal calcium uptake and release fluxes (*j*_*clc*_ and *j*_*tpc*_), including calcium leak fluxes (*j*_*clc, leak*_ and *j*_*tpc, leak*_); Na^+^/Ca^2+^ exchanger as principal mediator of calcium extrusion (I_NCX_); SR calcium reuptake through SERCA (J_SERCA_); and the transient outward K^+^ channel (I_to_) and rapid delayed rectifier K^+^ channel (I_Kr_), as main repolarization currents in mouse ventricular cardiomyocytes. Models were accepted in the WT population if they did not exhibit spontaneous calcium release in cytosolic calcium in any of our baseline protocols (CTRL, NAADP-AM, and ISO), resulting into a total of 423 accepted models, while covering a wide range of cellular profiles and biomarkers. The above acceptance criteria allowed us to investigate the role of lysosomal calcium handling under normal (nonproarrhythmic) physiological function, as well as to dissect its contribution under specific proarrhythmic conditions.

### Quantitative analysis of ionic and calcium features

Amplitude biomarkers were calculated as the absolute difference of maximum and minimum values. Boxplots are used to present the median (central line), 25th and 75th percentiles (box limits) of a distribution. Whiskers extend to the most extreme data points not considered as outliers, and outliers are depicted individually as independent data points. Fold changes of the calcium-related amplitudes were calculated from the medians of the populations being compared. For the analysis of arrhythmic events under calcium overload protocols, calcium transients with oscillations during the relaxation phase that could not be clearly ascribed to spontaneous calcium release events were excluded from our analysis.

## Results

### Model validation of lysosomal calcium handling

To investigate the role of lysosomal calcium handling on ventricular function, the mouse ventricular model of Morotti et al. (24) was extended to include a lysosomal compartment (see materials and methods). In brief, based on experimental evidence on organelle localization and function (13,18,19,29), lysosome action was modeled as an additional calcium pool, with function primarily driven by calcium loading from the junctional subspace, and NAADP-modulated release to the cytosol via TPC2 channels. The lysosome pool can thereby act as an additional calcium source depending on lysosomal calcium concentration, contributing to the CICR process.

Lysosome model calibration was conducted as follows. Without activating ligands, NAADP-modulated TPC2 openings are typically very brief, with open probability $P_{0}<0.0009$ (34). These were described by adopting a previously validated Gaussian distribution on NAADP concentration and an endogenous *[NAADP]* value of 1 nM (30), yielding an upper bound for $P_{0}$ of 0.016, close to the experimental findings. *[NAADP]* was increased to 15 nM when simulating fully opened TPC2 channels, resulting in a $P_{0}=0.0134$, close to the maximum open probability observed in experiments (34).

The magnitudes of lysosomal calcium fluxes were then calibrated based on experimental data from both WT and genetically modified TPC2-KO mouse cardiomyocytes (13). In these experiments (see Fig. 2), application of exogenous NAADP (NAADP-AM) increased calcium transient amplitude in WT but not in TPC2-KO cardiomyocytes. Simulated NAADP-AM action by increased *[NAADP]* resulted in a stronger calcium release from the lysosome compartment via TPC2, recapitulating the increase of cytosolic calcium amplitude (Fig. 2 *A*). When lysosomal calcium release ($J_{ls,rel}$) was set to zero (denoted as TPC2-KO) to mimic the genetically modified TPC2-KO cardiomyocytes, cytosolic calcium transient features were unaltered by an increase in *[NAADP]*, in agreement with the experimental data (Fig. 2 *B*).

Further validation of the lysosomal calcium handling model was conducted under additional pharmacological protocols exerting an action on the CICR process. Experimentally, β-adrenergic stimulation by ISO increased calcium transient amplitude in both WT and TPC2-KO, with the response of TPC2-KO significantly reduced compared with WT (Fig. 2 *C*–*D*). Both cardiomyocyte types also showed faster calcium transient relaxation in response to ISO. Likewise, the activation of the β-adrenergic response in the model by application of 100 nM ISO replicated these effects, with a larger response in WT.

### Lysosomal calcium release primarily modulates intracellular calcium handling by increasing SERCA reuptake and RyR release

In brief, in our modified model containing the lysosome as a compartment capable of taking up and releasing calcium, the calcium released from the lysosome enables an additional direct transport of calcium between the junctional and cytosolic spaces. In the absence of this transport mechanism, junctional calcium can only flow into the cytosol by pure intracellular diffusion, which is characterized by slower transport kinetics.

To investigate how these effects modulate intracellular calcium handling at the whole cellular level, a population of models approach was exploited to account for cellular variability in ionic currents and intracellular calcium fluxes, and their modulation of the full CICR process (26,27). To further enable investigations of the role of lysosomal calcium handling on arrhythmogenesis, only models characteristic of healthy physiological function (i.e., not exhibiting spontaneous calcium arrhythmic triggers in any of the described simulated pharmacological protocols) were retained in the population, giving a total of 423 accepted models (see materials and methods). Subsequent data are reported using this final population.

**Figure 3:**
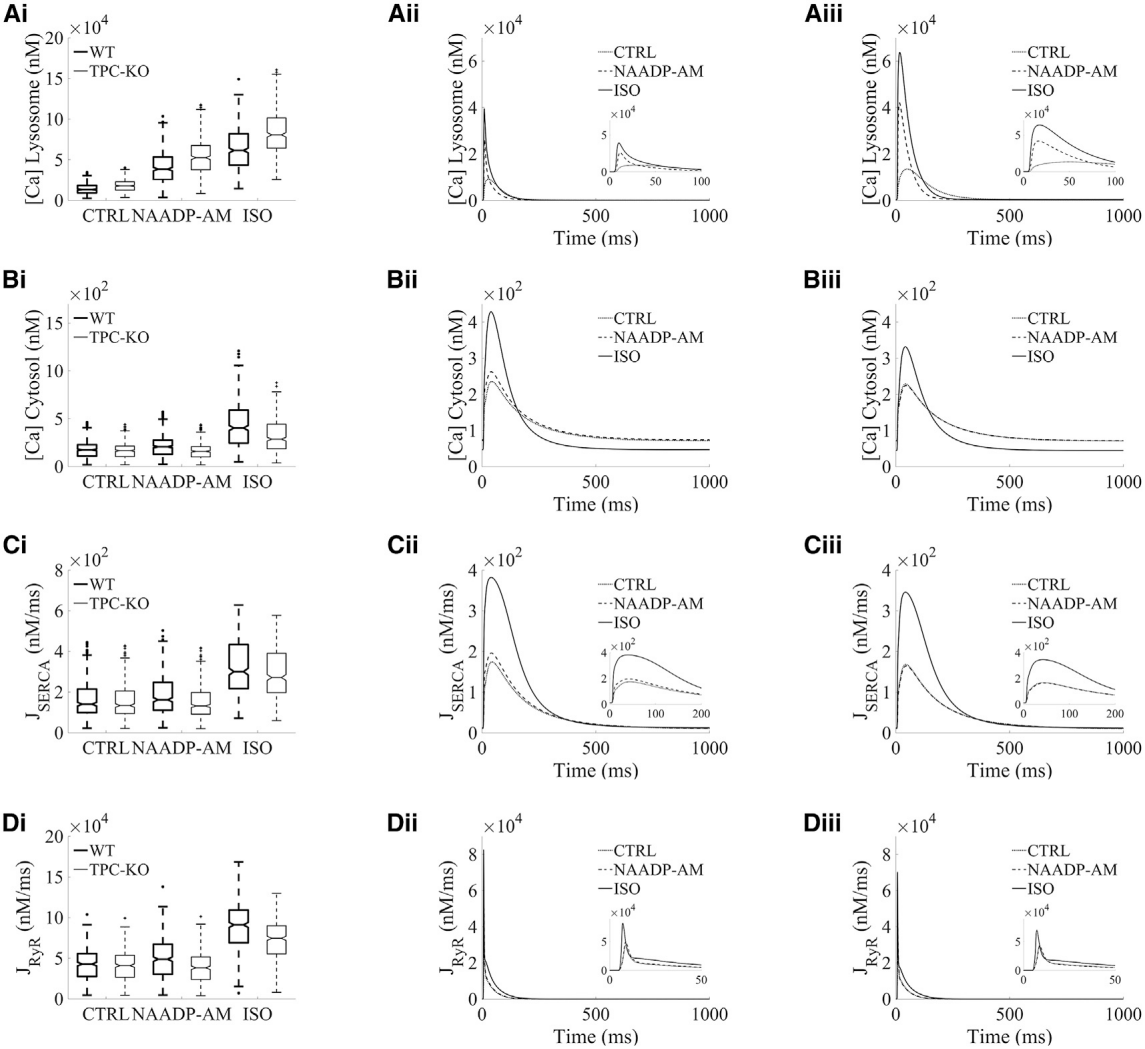
Lysosomal calcium release primarily modulates intracellular calcium handling by increasing SERCA reuptake and RyR release. Example selected traces for steady state are shown for: lysosomal calcium concentration (*A*i–iii), cytosolic calcium concentration (*B*i–iii), SERCA reuptake (*C*i–iii), and RyR release (*D*i–iii). For each quantity, the distribution of amplitudes under each experimental condition is shown in (i). Wild-type (WT) data are presented in black and TPC2 knockout (TPC2-KO) in red. The central line represents the median, boxes the interquartile range, and dotted lines the range. Each quantity is reported under control conditions (CTRL), in the presence of NAADP (NAADP-AM, 15 nM), and in the presence of β-adrenergic stimulation with isoprenaline (ISO) (100 nM). Representative traces of model outputs for WT and TPC2-KO are presented in (ii) and (iii), respectively (CTRL in *black*, NAAP-AM in *blue*, and ISO in *green*).

The boxplots in Fig. 3 show the amplitude distributions of calcium concentrations in the lysosome (Fig. 3 *A*i) and cytosolic compartments (Fig. 3 *B*i), and the intracellular calcium fluxes of SERCA reuptake (Fig. 3 *C*i) and RyR release (Fig. 3 *D*i) involved in intracellular calcium handling. Representative traces are shown for basal conditions (CTRL) and the investigated protocols of increased lysosomal function (NAADP-AM and ISO), as well as for WT versus TPC2-KO cardiomyocytes (Fig. 3, *A*ii–*D*ii and *A*iii–*D*iii). For CTRL, lysosomal calcium concentration was higher in TPC2-KO than WT as a result of the modeled genetic deletion of TPC in TPC2-KO cardiomyocytes (Fig. 3 *A*i–*A*iii), which precludes calcium from being released from the lysosomal compartment. No main median differences in cytosolic calcium amplitude were observed between WT and TPC2-KO (Fig. 3 *B*i–*B*iii) in CTRL, consistent with nonsignificant experimental differences in calcium transient amplitude between both cardiomyocyte types under basal conditions (13). Evaluation of SERCA and RyR amplitudes showed no main median differences in CTRL (Fig. 3, *C*i–*C*iii and *D*i–*D*iii).

Under application of exogenous NAADP (NAADP-AM protocol), the increased lysosomal calcium loading and the increased lysosomal calcium release led to a 1.19-fold amplitude increase of cytosolic calcium in WT compared with CTRL (Fig. 3 *B*i). This difference was also apparent in the representative traces of cytosolic calcium concentration (Fig. 3 *B*ii). In particular, these effects led to a systolic increase in cytosolic calcium and similar diastolic levels in WT cardiomyocytes under NAADP-AM compared with CTRL (Fig. 3 *B*ii), while the cytosolic calcium transient was unaltered upon NAADP-AM application in TPC2-KO cardiomyocytes (Fig. 3 *B*iii). Quantitatively, the calibrated population predicted a normalized increase in cytosolic calcium amplitude under NAADP-AM relative to CTRL of 0.200 ± 0.003 (mean ± standard error) in WT versus −0.035 ± 0.001 in TPC2-KO. These values are in agreement with the ranges of 0.16 ± 0.05 for WT and −0.06 ± 0.04 for TPC2-KO in experimental findings (13), further validating model predictions. Mechanistically, the increased cytosolic calcium loading under NAADP-AM in WT cells consequently promoted calcium reuptake by SERCA, causing a 1.15-fold increase in SERCA amplitude (Fig. 3 *C*i), which was also linked to a 1.14-fold increase calcium release by RyR, respectively, compared with the CTRL protocol (Fig. 3 *D*i). No main median differences with respect to basal conditions were observed in the amplitude of these fluxes for TPC2-KO cardiomyocytes in the comparison of NAADP-AM and CTRL protocols.

When β-adrenergic stimulation by ISO was simulated, the activation of PKA phosphorylation targets (I_CaL_, RyR, phospholamban, and phospholemman in our model) resulted in a higher lysosomal calcium load and release compared with the NAADP-AM protocol (Fig. 3 *A*i). Matching the experimental findings, there was also a greater response to ISO in WT cells than in TPC2-KO; ISO application increased the cytosolic calcium amplitude by 2.32-fold compared with CTRL for WT, and by 1.41-fold in WT versus TPC2-KO models in ISO (Fig. 3 *B*i). In terms of relative changes of cytosolic calcium amplitude, the population predicted increases of 1.439 ± 0.027 in WT and 0.898 ± 0.021 in TPC2-KO are in agreement with the ranges of 1.23 ± 0.24 and 0.57 ± 0.17 increase in experimental findings, respectively (13). ISO application led to a stronger 1.11-fold increase in calcium reuptake by SERCA in WT versus TPC2-KO (Fig. 3 *C*i) and a 1.22-fold increase of calcium release by RyR (Fig. 3 *D*i).

Altogether, the results presented in this section qualitatively and quantitatively recapitulate the findings associated with experimental observations of lysosomal function on the calcium transient in both WT and genetically modified TPC2-KO mouse cardiomyocytes (13). These results also illustrate an impact of lysosomal calcium in intracellular calcium handling by increasing the cytosolic calcium peak concentration, which in turn potentiates SERCA reuptake, increases the SR calcium load, and promotes CICR via RyR release.

### Lysosomal calcium release promotes spontaneous calcium release under hypercalcemia and β-adrenergic stimulation by increasing RyR open probability

Following our model validation and mechanistic findings of a lysosomal modulation of CICR under normal calcium loading, we further tested the model to investigate mechanisms of lysosomal involvement under proarrhythmic conditions.

Firstly, we simulated conditions of calcium overload by hypercalcemia (raising extracellular calcium from 1.0 to 1.8 mM) coupled with β-adrenergic stimulation. Only models exhibiting either normal calcium transients or clear spontaneous calcium events (i.e., excluding those with minor oscillatory behavior during relaxation) under this protocol were retained for analysis, leaving a total of 357 models. Out of the 357 cellular profiles (none exhibiting spontaneous release events under basal conditions), 52% of the models displayed spontaneous calcium release events in WT compared with 44% in TPC2-KO models, highlighting a lysosomal involvement in the triggering of intracellular calcium proarrhythmic events.

**Figure 4:**
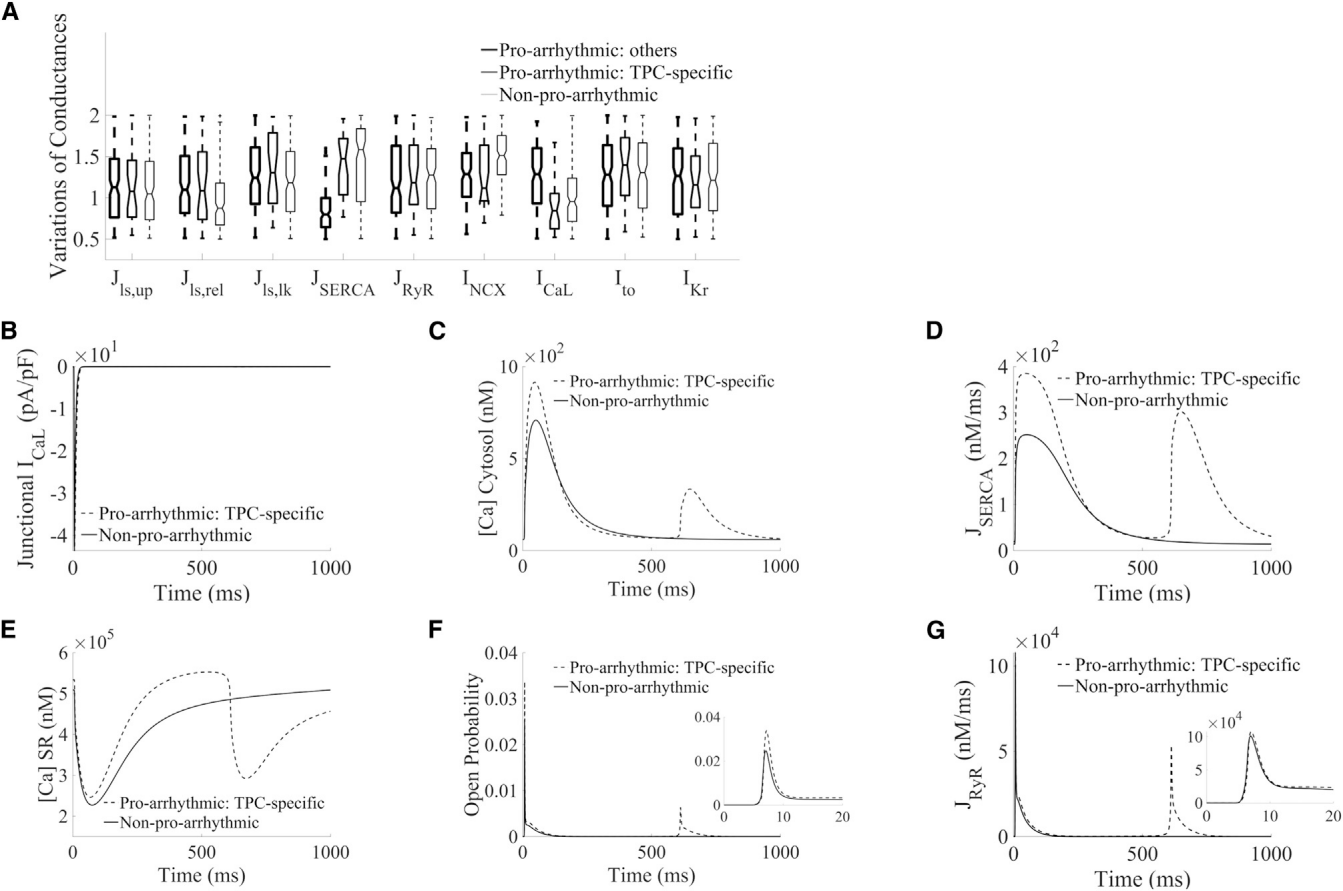
Ionic properties and calcium fluxes underlying spontaneous calcium events under hypercalcemia and sustained β-adrenergic stimulation. (*A*) Comparison of ionic properties of TPC-specific proarrhythmic models (*blue*), other proarrhythmic models (*red*), and nonproarrhythmic models (*magenta*) in WT. (*B*–G) Traces of junctional L-type calcium current, cytosolic and SR calcium concentrations, SERCA and RyR fluxes, and RyR open probability, respectively, comparing example selected TPC-specific proarrhythmic and nonproarrhythmic models in WT under similar I_CaL_ influx.

To better understand the mechanisms where lysosomal calcium handling is directly responsible for these potentially proarrhythmic events, TPC-specific proarrhythmic models (*n* = 30) were extracted from the population, meaning that the selected models exhibited spontaneous calcium release in WT (i.e., with active lysosomal TPC2 release) but not in their paired TPC2-KO configurations. The remaining proarrhythmic models featured spontaneous occurrence in both WT and TPC2-KO, while nonproarrhythmic models did not show spontaneous events in either WT or TPC2-KO. In terms of ionic properties underlying variability in cell-to-cell response, TPC-specific proarrhythmic profiles favored larger lysosomal calcium release than nonproarrhythmic models (J_ls,rel_, Fig. 4 *A*). Importantly, the TPC-specific proarrhythmic profiles were characterized by smaller densities in L-type calcium current compared with nonarrhythmic profiles (I_CaL_, Fig. 4 *A*).

Taken together, our findings highlight that, in TPC-specific proarrhythmic models with similar I_CaL_ influx than the nonproarrhythmic ones (Fig. 4 *B*), lysosomal calcium release provided sufficient additional calcium load to the cytosol to potentiate SERCA reuptake for SR loading (Fig. 4, *C*–*E*), which increased RyR open probability for RyR release (Fig. 4, *F* and *G*).

**Figure 5:**
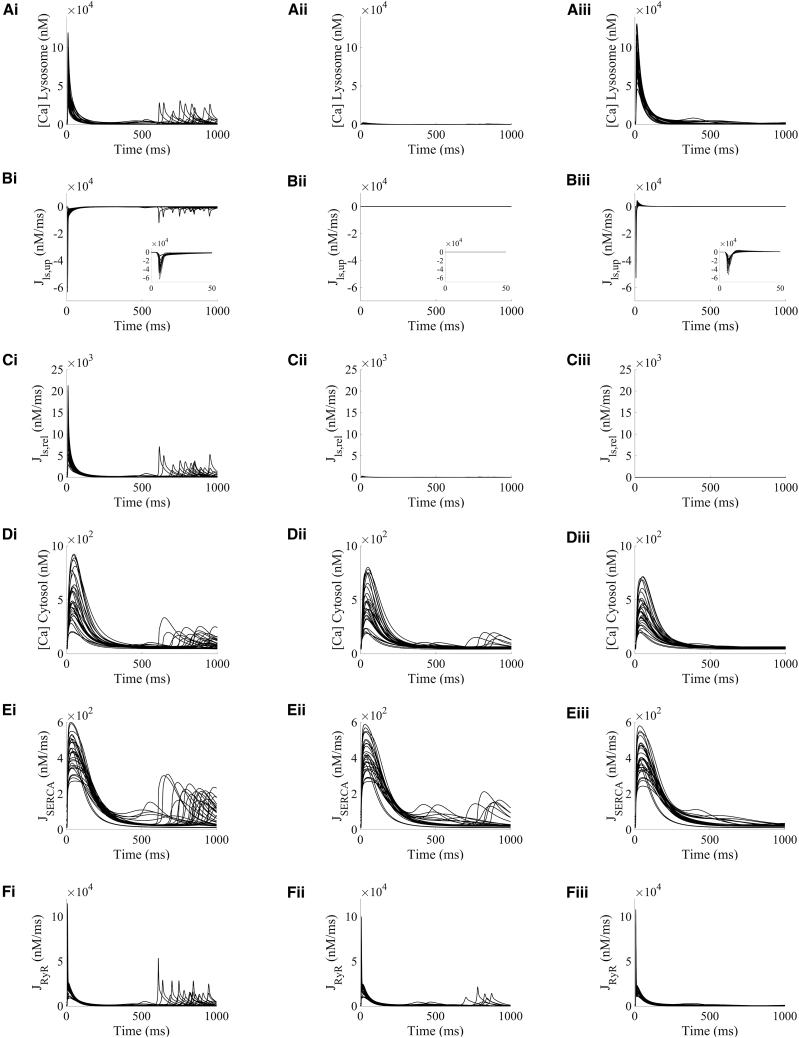
Lysosomal calcium release promotes spontaneous calcium release in TPC-specific proarrhythmic models under hypercalcemia and β-adrenergic stimulation by increasing RyR open probability. (*A*i–*F*i) Lysosomal calcium concentration, lysosomal uptake and release, cytosolic calcium concentration, and SR reuptake and release fluxes, respectively, under basal conditions. (*A*ii–*F*ii) Calcium concentrations and fluxes under lysosomal uptake block ($J_{ls,up}=0$). (*A*iii–*F*iii) Calcium concentrations and fluxes under lysosomal release block ($J_{ls,rel}=0$).

**Figure 6:**
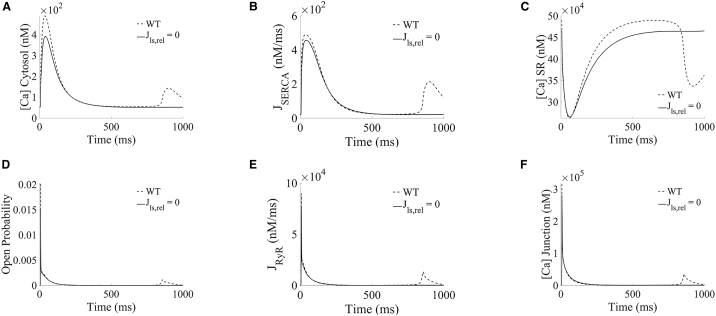
Loss of each lysosomal flux reduced spontaneous calcium release from SR in TPC-specific proarrhythmic models under hypercalcemia and β-adrenergic stimulation. (*A*–*F*) Example selected traces of cytosolic, SR and junctional calcium concentrations, SERCA and RyR fluxes, and RyR open probability, comparing scenarios in WT (*blue*) and blocking lysosomal calcium release ($J_{ls,rel}=0\text{,}$*magenta*).

A further analysis of lysosome modulation of spontaneous calcium release events is presented in Figs. 5 and 6. Fig. 5 illustrates the traces of the main calcium concentrations and fluxes involved in lysosomal calcium release under hypercalcemia and β-adrenergic stimulation for the TPC-specific proarrhythmic profiles, while Fig. 6 shows representative traces of under WT and TPC2-KO for further detailed mechanistic analysis. In the population of models, hypercalcemia resulted in lysosomal overload (Fig. 5 *A*i) compared with basal conditions (Fig. 3 *A*i), which contributed to a stronger calcium release from lysosome to cytosol (Fig. 5 *B*i). This was confirmed by an increase of total calcium reuptake by SERCA (Fig. 5 *C*i), leading to a higher SR calcium concentration and consequently increasing the likelihood of spontaneous events via RyRs (Fig. 5 *D*i).

Continuing to focus on models with TPC-dependent arrhythmias, the influence of lysosomal calcium handling in mediating spontaneous calcium release events was further dissected by blocking each of the respective lysosomal fluxes in the WT. The block of lysosomal calcium uptake ($J_{ls,up}$) precluded calcium loading in the lysosome compartment (Fig. 5 *A*ii), reducing spontaneous calcium release events to 37% (11/30) compared with TPC-specific proarrhythmic models in WT (Fig. 5 *D*ii). In terms of impact on function, the block of the lysosomal uptake flux impeded the loading of the lysosomal compartment, resulting in the loss of lysosomal calcium release (Fig. 5, *A*ii–*C*ii). The absence of the lysosomal calcium transport decreased calcium transient amplitude (Fig. 5 *D*ii) and thus calcium reuptake by SERCA (Fig. 5 *E*ii) and the magnitude of RyR release (Fig. 5 *F*ii).

On the other hand, loss of the lysosomal release in TPC2-KO cardiomyocytes (as mimicked by setting $J_{ls,rel}=0$) had a dual antiarrhythmic effect, resulting in the complete abolishment of spontaneous calcium release events in all TPC-specific proarrhythmic models. First, although the lysosome compartment now gets loaded (Fig. 5 *A*iii), the block of lysosomal release precludes its calcium load from flowing directly into the cytosol, getting transiently stored for a longer period of time in this compartment. The absence of the fast lysosomal pathway thus resulted in a similar antiarrhythmic mechanism as described above in the case of lysosomal uptake block (decreased calcium transient amplitude [Figs. 5 *D*iii and 6 *A*], reduced SERCA uptake [Figs. 5 *E*iii and 6 *B*], reduced SR concentration [Fig. 6 *C*], and, therefore, smaller RyR open probability [Fig. 6 *D*], smaller magnitude of RyR release [Figs. 5 *F*iii and 6 *E*], and smaller junctional loading [Fig. 6 *F*]). Second, a significant part of the lysosomal calcium concentration (approximately 86% in median) is reverted into the junctional space through the lysosomal loading channels, which is even noticeable in steady-state conditions (positive peak in Fig. 5 *B*iii). The additional calcium being released back into the junction contributes to reduce CICR, which reduced calcium loads in the cytosol, SR, and junction (Fig. 6, *A*, *C*, and *F*), drastically diminishing RyR open probability (Fig. 6 *D*), and therefore for spontaneous calcium release, when RyRs open.

In summary, approximately 16% (30/186) of our investigated models exhibiting spontaneous calcium release events under hypercalcemia and β-adrenergic stimulation were directly linked to lysosomal action due to an increased RyR open probability. Such lysosome-specific events can be effectively counteracted by inhibition of lysosomal calcium release, in agreement with experimental findings of decreased arrhythmic propensity in TPC2-KO mice (13).

**Figure 7:**
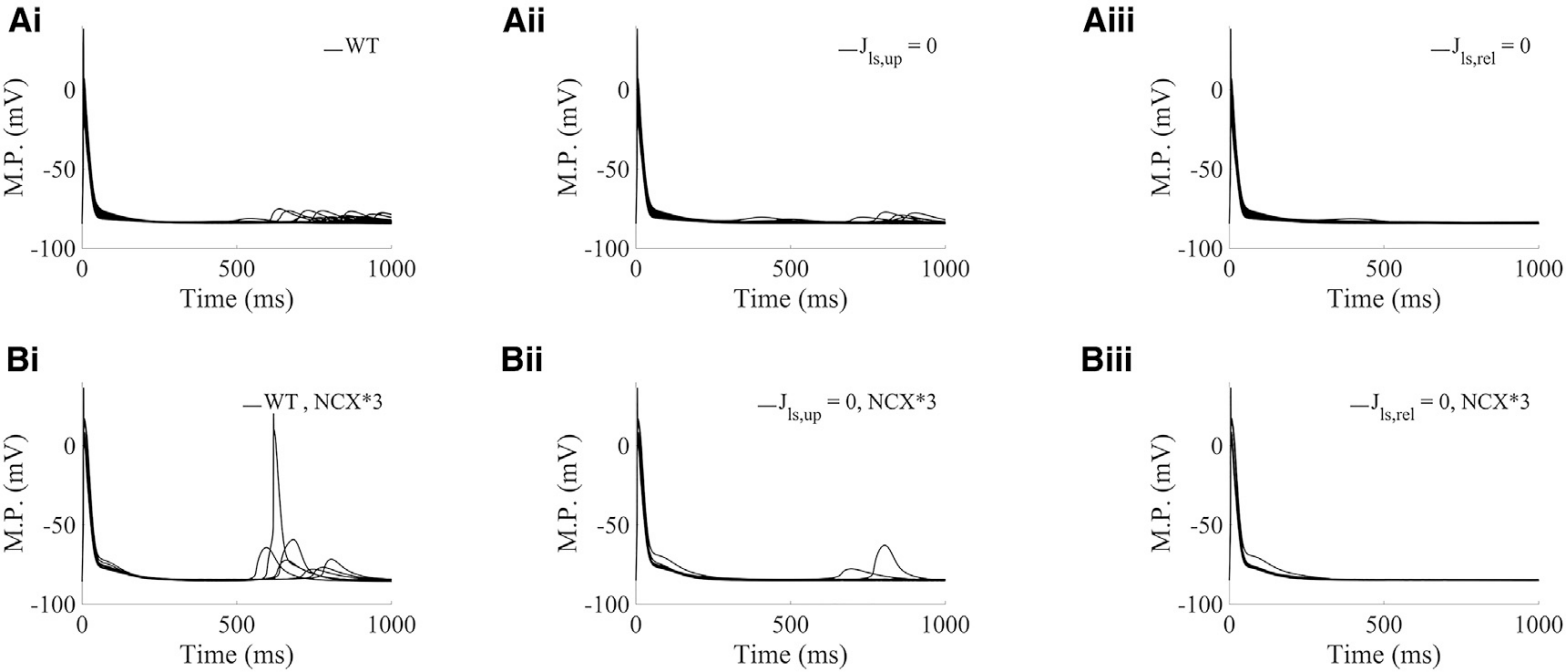
Lysosomal calcium release produces delayed afterdepolarizations by promoting spontaneous calcium release in TPC-specific proarrhythmic models under hypercalcemia and β-adrenergic stimulation. (*A*i–iii): Action potentials in the TPC-specific category with NCX current under basal variation (range of 0.5- to 2-fold changes), comparing scenarios in WT, blocking lysosomal calcium loading ($J_{ls,up}=0$), and blocking lysosomal calcium release ($J_{ls,rel}=0$), respectively. (*B*i-iii) Action potentials in the TPC-specific category when NCX current variation is increased by 3-fold (range of 0.5 × 3-fold to 2 × 3-fold changes), comparing scenarios in WT, blocking lysosomal calcium loading ($J_{ls,up}=0$), and blocking lysosomal calcium release ($J_{ls,rel}=0$), respectively. M.P., membrane potential.

The relationship of spontaneous calcium release events with delayed afterdepolarizations (DADs) at the action potential level is presented in Fig. 7. The spontaneous calcium release events mediated by lysosomal calcium release in TPC-specific proarrhythmic models activates the forward mode of NCX. This results in calcium extrusion through the membrane with an accompanying inward current, which elevates transmembrane voltage in the action potential as DADs (Figs. 5 *D*i and 7 *A*i) in comparison with the absence of spontaneous release events and DADs in such models under block of lysosomal release (Figs. 5 *D*iii and 7 *A*iii).

Since NCX current directly mediates DAD formation, we generated an additional population of models with enhanced NCX current by threefold to investigate its relationship with DAD amplitude. In the new population, 671 models were calibrated in CTRL, NAADP-AM, and ISO protocols by not exhibiting spontaneous calcium release events. Under hypercalcemia, we newly excluded for analysis those models with minor oscillatory behavior during relaxation that might not be clearly ascribed to spontaneous calcium release events. This exhaustive pruning led to a total of 7 TPC-specific models (compared with 30 TPC-specific models in basal variation), all exhibiting larger DADs than those in basal current variation (Fig. 7, *A*i and *B*i). We therefore find that an increase of NCX current directly contributes to DAD amplitude also in TPC-specific models (Fig. 7 *B*i), which can be successfully counteracted by block of TPC2 lysosomal release (Fig. 7 *B*iii).

### Lysosomal calcium release promotes spontaneous calcium release under fast pacing and β-adrenergic stimulation by increasing RyR open probability

Following experimental evidence of an involvement of lysosomal calcium handling in mediating arrhythmic events under chronic β-adrenergic stimulation and burst pacing (13), we hypothesized similar proarrhythmic mechanisms due to intracellular calcium accumulation by fast pacing as in concomitant hypercalcemia and sustained β-adrenergic stimulation. For these investigations, our populations of cardiomyocyte models were paced at 10 Hz for 150 beats to raise intracellular calcium, followed by a transition to slow pacing at 1 Hz for 5 consecutive beats, where spontaneous calcium release events were recorded.

From the original 423 models in the population, 8 were excluded for proarrhythmic analysis in fast pacing under the same criteria as aforementioned (oscillation in calcium transient not clearly ascribed to spontaneous calcium release). Out of the 415 calibrated cellular profiles, 59% of WT models displayed spontaneous calcium release events during their transitions from fast to slow pacing compared with 44% in TPC2-KO models, newly demonstrating a lysosomal contribution to intracellular calcium proarrhythmic events. TPC-specific proarrhythmic profiles (*n* = 60) were extracted from the new population, based on those exhibiting spontaneous calcium release events when transitioning from fast to slow pacing in WT, while not in their paired TPC2-KO configuration. In terms of ionic properties underlying cellular variability, TPC-specific proarrhythmic profiles were characterized by smaller I_CaL_ and I_NCX_ densities, together with smaller levels of SERCA reuptake (J_SERCA_) and RyR release (J_RyR_), respectively, compared with nonarrhythmic profiles (Fig. S1 *A*).

**Figure 8:**
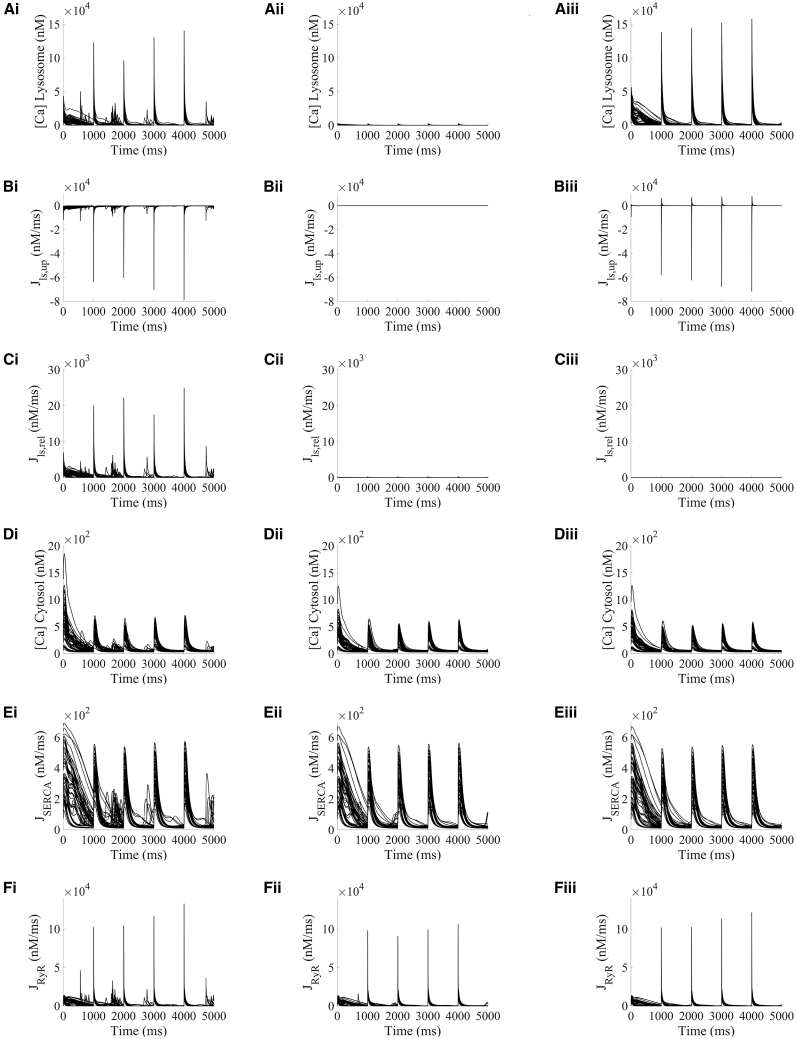
Lysosomal calcium release promotes spontaneous calcium release in TPC-specific proarrhythmic models under fast pacing and β-adrenergic stimulation by increasing RyR open probability. All results presented under initial fast pacing at 10 Hz. The transition from fast to slow pacing occurred at time 0. (Ai–*F*i) Lysosomal calcium concentration, lysosomal uptake and release, cytosolic calcium concentration, and SR reuptake and release fluxes, respectively, under basal conditions. (Aii–*F*ii) Calcium concentrations and fluxes under lysosomal uptake block ($J_{ls,up}=0$). (*A*iii–*F*iii) Calcium concentrations and fluxes under lysosomal release block ($J_{ls,rel}=0$).

The key intracellular concentrations and calcium fluxes involved in CICR are presented in Fig. 8 for the TPC-specific proarrhythmic models during their transition from fast to slow pacing. In WT, intracellular calcium accumulation due to the fast pacing portion of the protocol is apparent at the start of the transition (Fig. 8 *D*i) compared with basal conditions (Fig. 3 *B*i). This calcium overload is taken back up into the SR by SERCA (Fig. 8 *E*i), leading to subsequent SR overload and a persistent release by RyR in the form of spontaneous calcium events during the entire transition in pacing (Fig. 8 *F*i). Fast pacing model runs were repeated at a faster pacing rate of 25 Hz and analogous findings were observed (Fig. S3).

**Figure 9:**
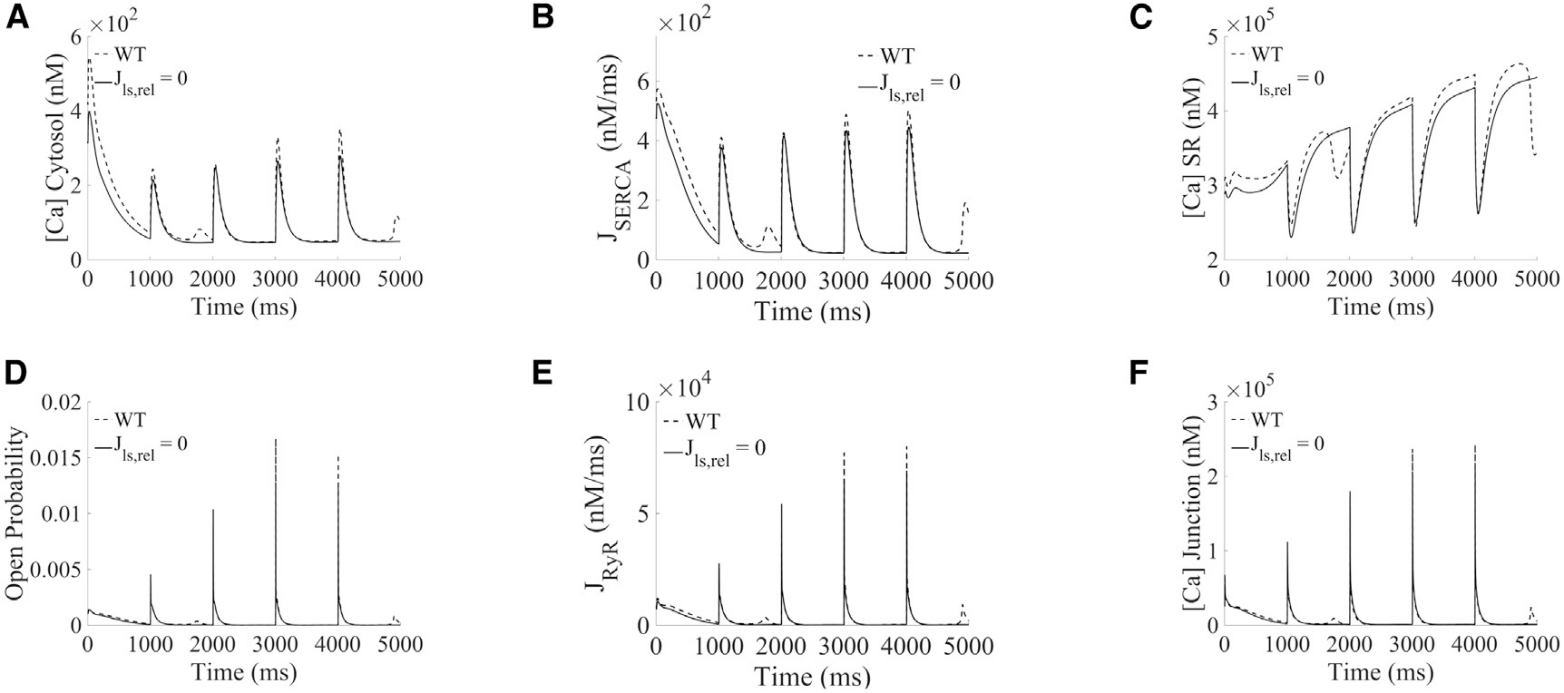
Loss of each lysosomal flux reduced spontaneous calcium release from SR in TPC-specific proarrhythmic models under fast pacing and β-adrenergic stimulation. All results presented under initial fast pacing at 10 Hz. The transition from fast to slow pacing occurred at time 0. (*A*–*F*) Example selected traces of cytosolic, SR and junctional calcium concentrations, SERCA and RyR fluxes, and RyR open probability, comparing scenarios in WT (*blue*) and blocking lysosomal calcium release ($J_{ls,rel}=0\text{,}$*magenta*).

The influence of lysosomal calcium handling in mediating these spontaneous calcium release events was investigated by blocking each of the respective lysosomal fluxes (Fig. 8, *middle and right columns*). While for the hypercalcemia case simulations were in a stable state, the transition from fast to slow pacing produced beat-by-beat changes in different models. Selected traces of a proarrhythmic TPC-specific model under WT and TPC-KO are presented in Fig. 9. Similar to the hypercalcemia case, block of the lysosomal release ($J_{ls,rel}=0$) was accompanied by an additional reversal of part of the lysosomal calcium concentration into the junctional space via the lysosomal loading channel (positive peaks in Fig. 8 *B*iii), and precluded calcium load in the lysosome (Fig. 8, *A*iii–*C*iii), leading to the abolition of the fast calcium pathway from the junctional space into the cytosol. This resulted into the previously discussed cascade of decreased cytosolic calcium transient amplitude (Figs. 8 *D*ii and 9 *A*), reduced SERCA uptake (Figs. 8 *E*ii and 9 *B*), decreased SR loading (Fig. 9 *C*), and smaller RyR open probability as driving force for RyR release (Figs. 8 *F*ii and 9, *D* and *E*) and reduced junctional loading (Fig. 9 *F*). Block of the lysosomal uptake ($J_{ls,up}=0$) decreased the incidence of spontaneous calcium release events to only 25% (15/60) of the TPC-specific proarrhythmic models. The same findings were obtained under faster pacing rates (Fig. S3).

## Discussion

In this study we present modifications to the well-established Morotti et al. cardiomyocyte model (24) to include a lysosomal calcium pool. To our knowledge, this work represents the first cardiac computational model to include a lysosomal compartment as part of beat-to-beat calcium signaling. Lysosomes, historically thought of as waste processing units, are now accepted to be complex signaling organelles. They are a store of calcium (35) and release calcium in response to the endogenous second-messenger NAADP (16). Work published from our group and others has confirmed that lysosomal calcium signaling is constitutively active in both ventricular (20,21) and atrial (12) cardiomyocytes and contributes to β-adrenergic responses (12,13,20,21,36). Furthermore, abolishing lysosomal calcium signaling, either pharmacologically or by genetic ablation of the NAADP effector channel TPC2, is protective against cardiac hypertrophy and arrhythmias (13,21). No cardiomyocyte model to date has sought to include lysosomal calcium signaling and we therefore set out to add a lysosomal calcium pool to a previously published, widely accepted model of mouse ventricular electrophysiology. This modified model was used to probe the impacts of lysosomal calcium signaling under both physiological and proarrhythmic conditions. After initial model construction, testing, and validation (Fig. 2), we used a population of models approach to generate a pool of models with a range of expression levels in other signaling proteins (e.g., I_CaL_, RyR, SERCA). Our model population was able to recapitulate experimental data: WT cells responded to NAADP with an increase in calcium transient amplitude, TPC2-KO cells did not (Fig. 3); TPC2-KO cells exhibited a reduced response to β-adrenergic stimulation by ISO (Fig. 3) and a reduced propensity to arrhythmogenesis under proarrhythmic conditions (Figs. 5 and 8). The major results of our simulation studies are consistent with a role for lysosomes in increasing calcium uptake to the SR by SERCA, release from the SR by RyRs, and RyR open probability (Figs. 4 and S1). The overall effect is to increase calcium release from the SR during CICR. In a small fraction of the models in our population, lysosomal calcium was found to be the direct cause of arrhythmogenesis under proarrhythmic conditions. Further consideration of these models showed that they did not exhibit spontaneous lysosomal release but DADs arising as a result of RyR-mediated spontaneous calcium release from the SR. Our further trials (Figs. 6, 9, and S3) suggested that, when the balance of other signaling effectors is appropriate, lysosomes acting as a fast transport can effectively shunt the location of calcium to promote DAD formation.

The Morotti et al. model (24) was chosen for modification to include the lysosomal calcium pool because it is closest in nature to our published laboratory research (13); it is a murine ventricular cell model detailing both calcium and action potential characteristics. The Morotti et al. model also includes validated β-adrenergic and CaMKII signaling modules, both of which have been implicated in NAADP-mediated signaling in the heart (13,37). The updated model presented in this paper was validated using published murine single-cell and ex vivo tissue data from our group (13) with the position of the lysosomal calcium pool justified by our structural studies (19). The lysosomal pool features calcium uptake, calcium release, and calcium leak. The calcium release mechanism is via TPC2 channels, with the TPC2 open probability in response to NAADP modeled on the basis of data from Penny et al. (30). NAADP-AM (15 nM) was used throughout our study as the NAADP concentration equivalent to nearly maximal TPC2 open probability.

The addition of the lysosomal calcium pool to our model also included a calcium leak channel and a calcium uptake mechanism. Although the candidate protein for neither of these processes has been identified, it is justified to include them here: calcium leak must occur from lysosomes as breakdown of the lysosomal hydrogen ion gradient after treatment with bafilomycin-A1 leads to a rundown of lysosomal calcium in the absence of other stimulation (35,38). Similarly, there must be a calcium uptake mechanism, as the lumenal calcium of lysosomes is several orders of magnitude higher than the cytosolic concentration (35). In the absence of characterized mechanisms for this process we adopted an analogous formulation to TPC2 channels. This correctly recapitulated our calcium transient data under different pharmacological protocols. This modeling assumption can nevertheless be easily revisited, should additional data sets on lysosomal calcium loading become available.

The study presented in this paper used a population of models approach to take into account that cells are not a homogeneous population and may respond to differing degrees based on inherent expression/activity level of various known calcium signaling and electrophysiology components. To this end, Latin hypercube sampling was used to create a population of 1000 models to represent a wide combination of underlying contributions. Of these, a subpopulation of 423 was accepted into the final population as behaving within the physiological envelope of our experimental data; they were not arrhythmic under any “physiological” conditions. In our simulations using this population of models, lysosomal calcium release primarily modulated intracellular calcium handling by increasing SERCA reuptake and subsequently RyR release. This is consistent with published data that suggest that stimulation of lysosomal calcium release with NAADP leads to an increase in SR calcium load, as measured by caffeine transient (12,20). The underlying action of the lysosomal calcium pool was essentially to act as a fast transport, moving calcium into the space from which SERCA pumps calcium into the SR. In comparison with WT, TPC2-KO models exhibited reduced cytosolic calcium concentration, SR calcium uptake (J_SERCA_), and RyR calcium release (J_RyR_) upon application of NAADP or ISO.

Our published work in ventricular cardiomyocytes and ex vivo cardiac tissue suggest that TPC2-KO is protective against the development of cardiac arrhythmias under conditions of acute or chronic high-dose β-adrenergic stimulation (13). This is also true of acute ISO exposure in single cells where TPC2 channels have been inhibited pharmacologically (21). As an analogous set of experiments, we exposed the population of 423 accepted models to proarrhythmic conditions in both WT and TPC2-KO configurations. Synergetic to β-adrenergic stimulation, these conditions were either increased extracellular calcium or rapid burst pacing, both of which are common proarrhythmic interventions in experimental studies of calcium overload. Similar to published data, we found that TPC2-KO is protective against the genesis of arrhythmia: 52% of WT models exhibited spontaneous events under β-adrenergic stimulation and high calcium, but this fell to 44% in TPC2-KO. Moreover, 59% of WT models exhibited spontaneous events under β-adrenergic stimulation and fast pacing, but this fell to 44% in TPC2-KO.

In a subpopulation of the accepted models exposed to proarrhythmic conditions, the WT model was found to be arrhythmic and the corresponding TPC2-KO was not. That is to say, the only difference between the rhythmic and arrhythmic simulations was the presence of lysosomal calcium signaling. These models comprised *n* = 30 for raised extracellular calcium with β-adrenergic stimulation and *n* = 60 for rapid pacing and β-adrenergic stimulation, or 16% (30/186) and 25% (60/243) of all models which exhibited arrhythmia for each proarrhythmic condition, respectively. We looked further at these TPC2-dependent arrhythmia models to try to determine what the action(s) of the lysosome might be under these circumstances, and therefore by which specific mechanism lysosomal calcium might be contributing to the genesis of rhythm disturbance under our proarrhythmic conditions, and/or in what way TPC2-KO is protective against arrhythmia. Analysis of the intracellular calcium traces during the genesis of spontaneous activity showed a profile of DADs arising from spontaneous SR calcium release (Figs. 5 and 8). In other cell types, from various species, lysosomal calcium release can lead to a cellular calcium transient, or cellular calcium oscillations, directly by recruiting CICR from the reticular store (16,39,40). There were, however, no instances in which we observed something akin to spontaneous lysosomal calcium release leading directly to a cytosolic calcium transient. This is consistent with published experimental work, which demonstrates the role of lysosomal calcium signaling in cardiomyocytes as increasing the size of the normal, electrically stimulated calcium transient as opposed to generating de novo transients by CICR (12,13,20). Although Nebel et al. reported the generation of a spontaneous signal on patch application of NAADP (21), we have not observed any similar phenomena through application from the patch Pipette (unpublished data), ultraviolet photolysis of a caged compound (12,13,20) or via the membrane using an esterified form (12,13). The results seen in our simulation study therefore also support our published experimental findings.

One way to consider the specific effects of lysosomal fluxes on models exhibiting TPC2-dependent arrhythmias is to investigate the consequence of removing lysosomal calcium contributions, flux-by-flux, from the WT versions of the models in question. Removing these fluxes from the models with TPC2-dependent arrhythmias (Figs. 6 and 9), regardless of the specific proarrhythmic condition, led to decreases in SERCA calcium flux, RyR calcium flux, SR calcium content, and RyR open probability. Taken together, this would suggest a conclusion consistent with that seen in our physiological signaling study: the presence of lysosomal calcium release boosts SR calcium content and release by acting as a fast transport. Furthermore, under particular proarrhythmic conditions, the shifting of calcium can occur to the level where the cell becomes at risk of DADs. This mechanism would also be consistent with published cellular recordings that showed a significant increase in SR calcium content, as measured by caffeine transient, after exposure to NAADP (12,20). This supports the conclusion that lysosomal calcium in TPC2-dependent arrhythmia models is acting to push those otherwise physiological cells into the arrhythmic category via actions related to SR calcium signaling.

In conclusion, we have modified an existing cellular model of cardiomyocyte electrophysiology and calcium handling to include a novel calcium pool, the lysosome. This is the first cardiac cellular model to include lysosomal calcium in beat-to-beat regulation. The lysosomal calcium pool, which was unknown at the turn of the century, is now understood to contribute to beat-to-beat calcium handling, response to β-adrenergic stimulation, and potentially to arrhythmogenesis. Our simulations support a role for the lysosome as a fast transport, acting ultimately to shunt calcium into the SR. The population of models approach allowed us to investigate a range of conditions and support that removing lysosomal calcium signaling by TPC2-KO reduces the likelihood of developing spontaneous activity under proarrhythmic conditions. Taken another way, our model suggests that there are some conditions under which lysosomal calcium signaling can be proarrhythmic. This modeling work leads to an important next experimental step: to investigate what these proarrhythmic conditions are in both healthy cells and those in disease states associated with an increased likelihood of arrhythmia.

### Limitations of the study

Our approach for lysosomal calcium handling modeling effectively considers the lysosomes as a separate compartment, feeding from the junctional space (shared by I_CaL_ channels and RyRs) and releasing their calcium load into the cytosol. This, together with appropriate considerations of lysosomal volume size, allows the lysosomal compartment to achieve high local calcium concentrations compared with the cytosol. Given that the cytosolic space is shared by CaMKII and phospholamban/SERCA, our lysosomal compartment can therefore be conceptually interpreted as in close proximity to these proteins, with lysosomal calcium release via TPC2 being able to directly modulate their function. Should sufficient evidence become available, the proposed modeling could be expanded to consider different (nano)domain configurations.

Previous studies (13) have shown that TPC2-KO reduced arrhythmic propensity under adrenergic stress conditions, and that calcium transient amplitude was reduced when blocking lysosomal release. Although these findings were replicated by our model, the reduced calcium transient could also have knock on effects on other downstream targets, not directly described in the model formulation. In addition, pharmacological observations (13) have also shown that CaMKII is required for responses to NAADP in ventricular myocytes from guinea pigs and mice. Although CaMKII is accounted for in the Morotti et al. model, we did not investigate in detail its interactions with lysosomal function. We hope to address these aspects in future research.

## Author contributions

Conceptualization, Z.M., R.A.B.B., and A.B.-O.; design and methodology, Z.M., R.A.B.B., and A.B.-O.; software, Z.M.; investigation, Z.M. and A.B.-O.; formal analysis, Z.M.; writing – original draft, Z.M.; writing – review & editing, Z.M., R.A.C., S.J.B., A.G., R.P., R.A.B.B., and A.B.-O.; contribution to theoretical development, R.A.C.; results discussion, S.J.B.; theoretical discussions, E.B., S.d.J., and R.P.; early model development, E.B., S.d.J. and R.P.; mechanistic discussions, A.G.; resources, R.A.B.B. and A.B.-O.; supervision R.A.B.B. and A.B.-O.

## Data Availability

All codes underpinning this research are openly available at: https://github.com/zhaomeng132/Ls_mv.

## Supporting material

Document S1. Figures S1–S3

Document S2. Article plus supporting material
