# Supplementary material for: Lysosomal calcium loading promotes spontaneous calcium release by potentiating ryanodine receptors
Source: Biophys J. 2023 Jun 15;122(15):3044–59. doi: 10.1016/j.bpj.2023.06.007 (PMC10432190; doi:10.1016/j.bpj.2023.06.007)
Supplement: Document S1. Figures S1–S3 [file mmc1.pdf]

Biophysical Journal, Volume *122*

Supplemental information

Lysosomal calcium loading promotes spontaneous calcium release by potentiating ryanodine receptors

Zhaozheng Meng, Rebecca A. Capel, Samuel J. Bose, Erik Bosch, Sophia de Jong, Robert Planque, Antony Galione, Rebecca A.B. Burton, and Alfonso Bueno-Orovio

Lysosomal calcium loading promotes spontaneous calcium release by potentiating ryanodine receptors

Zhaozheng Meng[1], Rebecca A Capel[1], Samuel J Bose[1], Erik Bosch[2], Sophia de Jong[2], Robert Planque[2], Antony Galione[1], Rebecca AB Burton[1*], Alfonso Bueno-Orovio[3*]

[1] Department of Pharmacology, University of Oxford, Oxford, UK

[2] Department of Mathematics, Vrije Universiteit Amsterdam, Netherlands

[3] Department of Computer Science, University of Oxford, Oxford, UK

address(es) of correspondence:

alfonso.bueno@cs.ox.ac.uk and rebecca.burton@pharm.ox.ac.uk

Running title: Modelling lysosomal calcium signalling in cardiac cells

Supplementary Material

Parameters		Units	Value	Ref.
**Volume**	Total cell (V_{tot})	pL	33	[1]
	Lysosome (V_{ls})	pL	2% V_{tot}	[2, 3]
**TPC**	Maximum open probability (P_{Omax})		0.014	[2, 4]
	Variance (P_{Osd}^2)		2.25	this study
	Mean (P_{Omean})		23	[2]
	Leak rate ($j_{clc,leak}$, $j_{tpc,leak}$)	s^{-1}	1.13E-05	this study
**Lysosome**	TPC flux density into junction (j_{clc})	s^{-1}	9.708	this study
	TPC flux density into cytosol (j_{tpc})	s^{-1}	12.135	this study
	[*NAADP*] for CTRL protocol	nM	1	this study
	[*NAADP*] for NAADP-AM protocol	nM	15	this study
	[*NAADP*] for ISO protocol	nM	15	this study
	Diffusion flux rate to junction ($J_{ls,j}$)	um^3/s	1.4219E-15	this study
	Diffusion flux rate to cytosol ($J_{ls,i}$)	um^3/s	1.3858E-15	this study

Table S1. Baseline parameters for the lysosome compartment.

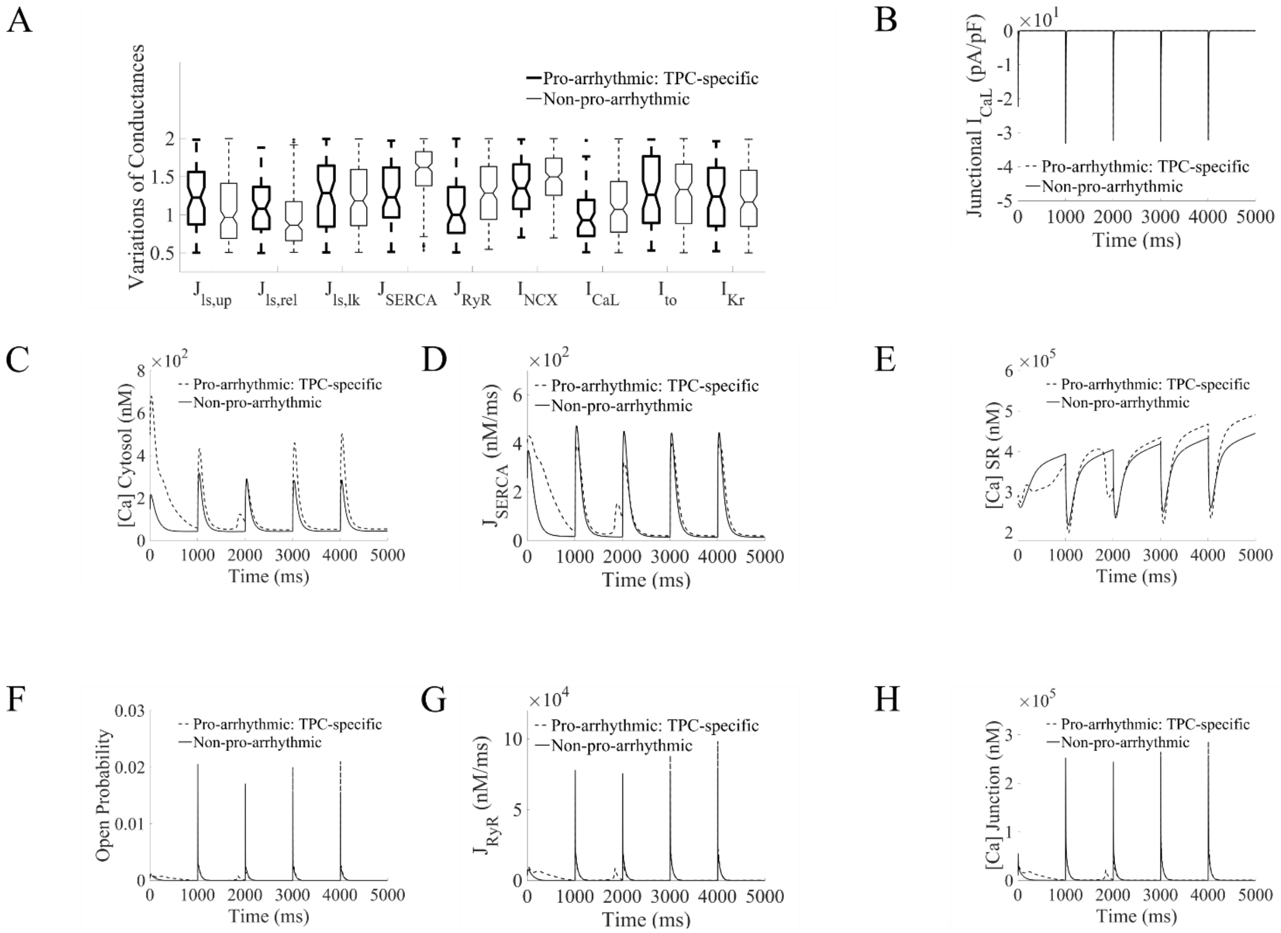

Figure S1. Ionic properties and calcium fluxes underlying spontaneous calcium events under fast pacing at 10 Hz and sustained β-adrenergic stimulation. **S1A:** Comparison of ionic properties of TPC-specific proarrhythmic models (blue), and non-proarrhythmic models (magenta) in WT. **S1B-S1H:** Traces of junctional L-type calcium current, cytosolic, SR and junctional calcium concentrations, SERCA and RyR fluxes, and RyR open probability, comparing TPC-specific pro-arrhythmic models, and non-proarrhythmic models in WT.

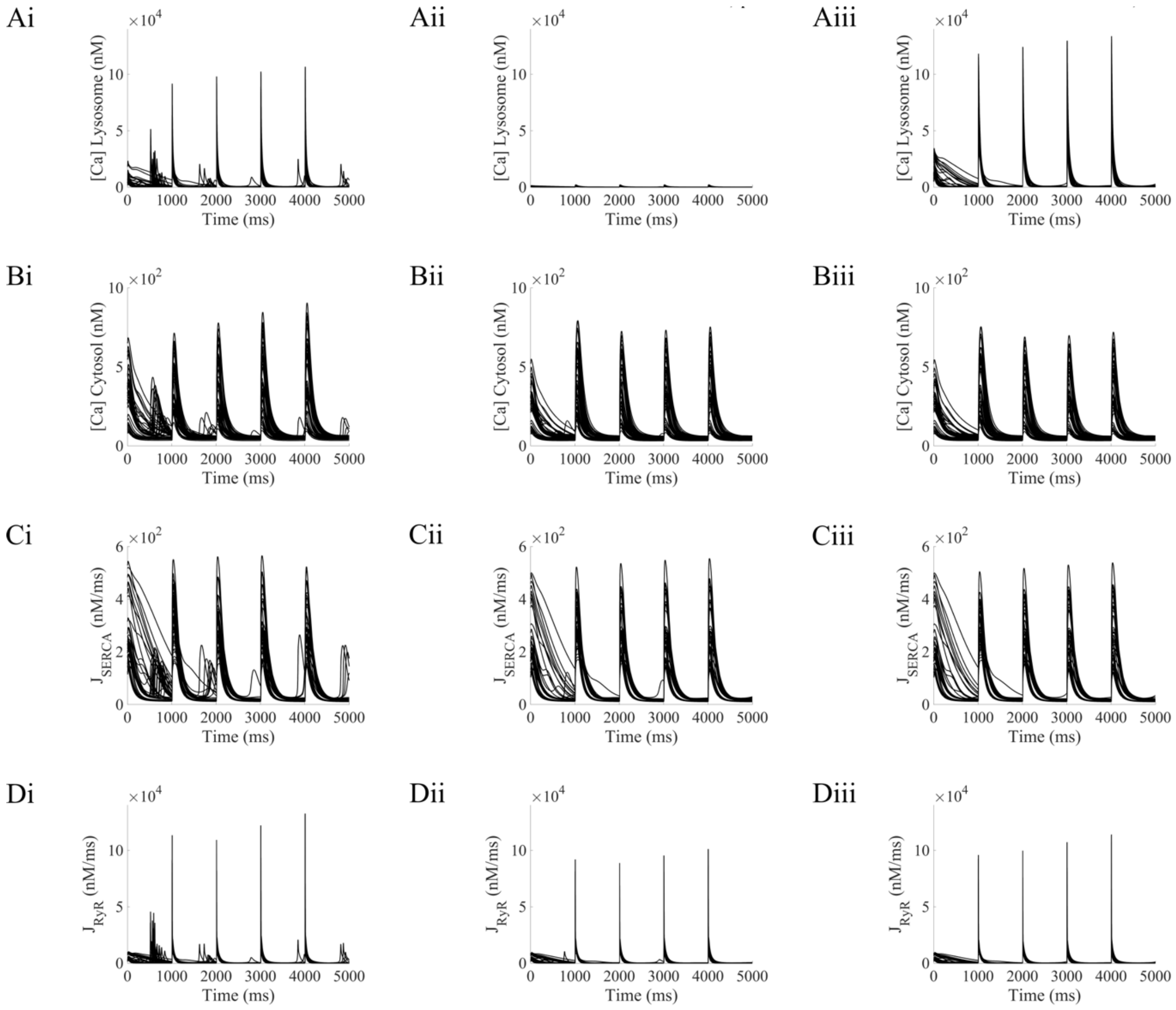

Figure S2. Lysosomal calcium release promotes spontaneous calcium release in TPC-specific pro-arrhythmic profiles under fast pacing and β-adrenergic stimulation, by increasing the junctional-SR calcium gradient. **S2Ai-S2Di:** Lysosomal calcium concentration, cytosolic calcium concentration, and SR reuptake and release fluxes, respectively, in basal conditions. **S2Aii-S2Dii:** Calcium concentrations and fluxes under lysosomal uptake block ($J_{ls,up} = 0$). **S2Aiii-S2Diii:** Calcium concentrations and fluxes under lysosomal release block ($J_{ls,rel} = 0$). All results presented under preliminary fast pacing at 25 Hz.

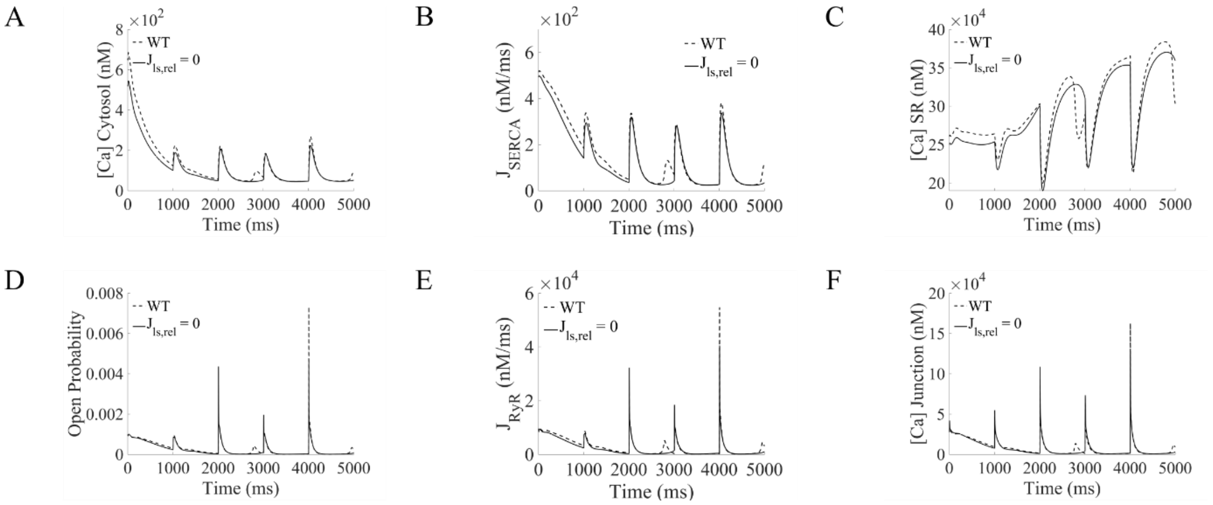

Figure S3. Loss of each lysosomal flux reduced spontaneous calcium release from SR in TPC-specific pro-arrhythmic models under fast pacing and β-adrenergic stimulation. **S3A-S3F:** Traces of cytosolic, SR and junctional calcium concentrations, SERCA and RyR fluxes, and RyR open probability, comparing scenarios in WT (blue), and blocking lysosomal calcium release ($J_{ls,rel} = 0,$ magenta). The beats presented in the results are at 1Hz following 25Hz fast pacing protocol.

Supplemental References

1. Morotti, S., et al., *A novel computational model of mouse myocyte electrophysiology to assess the synergy between Na+ loading and CaMKII.* Journal of Physiology-London, 2014. **592**(6): p. 1181-1197.
2. Penny, C.J., et al., *A computational model of lysosome-ER Ca2+ microdomains.* Journal of Cell Science, 2014. **127**(13): p. 2934-2943.
3. Aston, D., et al., *High resolution structural evidence suggests the Sarcoplasmic Reticulum forms microdomains with Acidic Stores (lysosomes) in the heart.* Sci Rep, 2017. **7**: p. 40620.
4. Pitt, S.J., et al., *TPC2 Is a Novel NAADP-sensitive Ca2+ Release Channel, Operating as a Dual Sensor of Luminal pH and Ca2+.* Journal of Biological Chemistry, 2010. **285**(45): p. 35039-35046.
